# Serum Lipids and Breast Cancer Risk: A Meta-Analysis of Prospective Cohort Studies

**DOI:** 10.1371/journal.pone.0142669

**Published:** 2015-11-10

**Authors:** Haibo Ni, Huixiang Liu, Rong Gao

**Affiliations:** Department of Neurosurgery, The First People’s Hospital of Zhangjiagang City, Suzhou, Jiangsu, China; University of South Alabama Mitchell Cancer Institute, UNITED STATES

## Abstract

**Purpose:**

Epidemiologic studies exploring causal associations between serum lipids and breast cancer risk have reported contradictory results. We conducted a meta-analysis of prospective cohort studies to evaluate these associations.

**Methods:**

Relevant studies were identified by searching PubMed and EMBASE through April 2015. We included prospective cohort studies that reported relative risk (RR) estimates with 95% confidence intervals (CIs) for the associations of specific lipid components (i.e., total cholesterol [TC], high-density lipoprotein cholesterol [HDL-C], low-density lipoprotein cholesterol [LDL-C], and triglycerides [TG]) with breast cancer risk. Either a fixed- or a random-effects model was used to calculate pooled RRs.

**Results:**

Fifteen prospective cohort studies involving 1,189,635 participants and 23,369 breast cancer cases were included in the meta-analysis. The pooled RRs of breast cancer for the highest versus lowest categories were 0.96 (95% CI: 0.86–1.07) for TC, 0.92 (95% CI: 0.73–1.16) for HDL-C, 0.90 (95% CI: 0.77–1.06) for LDL-C, and 0.93 (95% CI: 0.86–1.00) for TG. Notably, for HDL-C, a significant reduction of breast cancer risk was observed among postmenopausal women (RR = 0.77, 95% CI: 0.64–0.93) but not among premenopausal women. Similar trends of the associations were observed in the dose-response analysis.

**Conclusions:**

Our findings suggest that serum levels of TG but not TC and LDL-C may be inversely associated with breast cancer risk. Serum HDL-C may also protect against breast carcinogenesis among postmenopausal women.

## Introduction

Cancer and cardiovascular diseases are the leading causes of death in developed countries, and they are expected to be the most important determinants of death in developing countries by 2030 [1]. Lifestyle and dietary factors such as obesity and high-fat diets are frequently incriminated as common risk factors of these diseases. While unfavorable lipid profiles, as a result of unhealthy diet as well as physical inactivity, have long been implicated in cardiovascular diseases [2], the effect of dyslipidemia on breast cancer incidence remains unclear.

Cholesterol plays important roles in cellular structure and function and as an obligatory precursor to several biochemical pathways, especially the synthesis of steroid hormones, which are implicated in the etiology of breast cancer [3, 4]. Since the 1980s, a number of epidemiologic studies [5–19] that investigated the relationships between lipid components and breast cancer risk have reported contrasting results of both inverse and positive associations. A recent meta-analysis [20] that was largely based on case-control studies yielded 8% (non-significant) and 39% increased risks of breast cancer in relation to higher triglycerides (TG) and lower high-density lipoprotein cholesterol (HDL-C), respectively. However, the quality and strength of evidence was limited because including case-control studies inevitably introduced selection and recall biases. Moreover, several large cohort studies [7, 9, 11, 17] that did not dichotomize TG or HDL-C levels were not included. The effects of other lipid components such as total cholesterol (TC) and low-density lipoprotein cholesterol (LDL-C) on the incidence of breast cancer also remain to be determined.

Therefore, the objective of this meta-analysis was to systematically evaluate the association between individual lipid components and breast cancer risk in prospective cohort studies.

## Materials and Methods

### Literature search

We performed literature searches of the PubMed and EMBASE databases without restrictions through April 2015. The following terms were used: “lipid,” “lipoprotein,” “cholesterol,” “triglyceride,” “dyslipidemia,” “breast neoplasms,” “breast cancer,” “risk,” “incidence,” and “prevalence.” Moreover, we reviewed the reference lists of retrieved articles for additional studies. We followed the MOOSE guidelines to conduct and report this meta-analysis.

### Study selection

Studies were eligible for this meta-analysis if they fulfilled the following criteria: 1) the study design was a prospective cohort study, 2) the exposure of interest was serum concentration of at least one of the selected lipid components (TC, HDL-C, LDL-C, TG) measured prior to breast cancer diagnosis, 3) the outcome of interest was the occurrence of breast cancer, and 4)the relative risk (RR) with corresponding 95% confidence interval (CI, or data to calculate them) were reported. If data were duplicated in more than one study, we included the study with the largest number of cases.

### Data Extraction and Quality Assessment

Two investigators (H.B.N. and H.X.L.) independently reviewed the articles and extracted the data from all eligible publications. Any disagreement was settled by discussion. The following data were recorded: first author’s surname, publication year, country of origin, ethnicity, study and follow-up periods, characteristics of study population (age and menopausal status), numbers of cases and participants, ranges of serum lipid levels, RRs from the most fully adjusted model for the highest versus lowest category of serum lipids and the corresponding 95% CIs, and matching or adjustments for confounding factors.

The methodological quality of included studies was assessed by the nine-star Newcastle-Ottawa Scale (NOS) [21], which consists of three major aspects: selection, comparability, and exposure or outcome. A study with 7 or more stars is considered to be high quality.

### Statistical Analysis

RR was used as a common measure of association, and the hazard ratios was deemed equivalent to RRs because the absolute risk of breast cancer is quite low. From each study, we extracted the risk estimates that reflected the comparison of the highest versus lowest categories and the greatest degree of control for potential confounders. Those articles providing results stratified by menopausal status only were treated as two separate reports [7, 8]. For studies that did not use the category with the lowest concentration as the reference [9–12], we recalculated RRs using the method described by Hamling et al [22].

The homogeneity of RRs among studies was assessed with Cochran’s Q statistic (P < 0.1 was deemed statistically significant) and quantified using the *I* ^2^ statistic [23]. When substantial heterogeneity was found, we used a random-effect model to calculate the summary risk estimates; otherwise, the fixed-effect model was adopted [24]. Prespecified subgroup analyses based on geographic area, ethnicity, follow-up length, number of cases, menopausal status, and adjustment for dietary factors were performed to assess the impacts of these variables on outcomes. Due to limited number of eligible studies, subgroup analyses were not performed for LDL-C. We further conducted sensitivity analysis by excluding low-quality studies to examine the robustness of the combined risk estimates. We also investigated the impact of a single study on the overall results by omitting one study in each turn.

We next conducted dose-response relationships of serum lipids with breast cancer risk based on the method proposed by Greenland and Longnecker [25] and Orsini et al [26]. Only the studies that reported the number of cases, person-years of follow-up, and adjusted RRs with 95% CIs for at least three quantitative exposure categories were included. Because two main studies did not report numbers of cases by different categories [13, 18], another study [27] that had overlapping data was retrieved for this analysis. Because of sparse data on LDL-C, dose-response analysis was only performed on TC, HDL-C, and TG.

Potential publication bias was evaluated with Begg’s rank correlation tests [28] and Egger’s linear regression tests [29]. All analyses were performed using STATA version 11.0 (StataCorp, College Station, TX, USA). P < 0.05 was considered statistically significant, except where otherwise specified.

## Results

### Literature search

**Fig 1:**
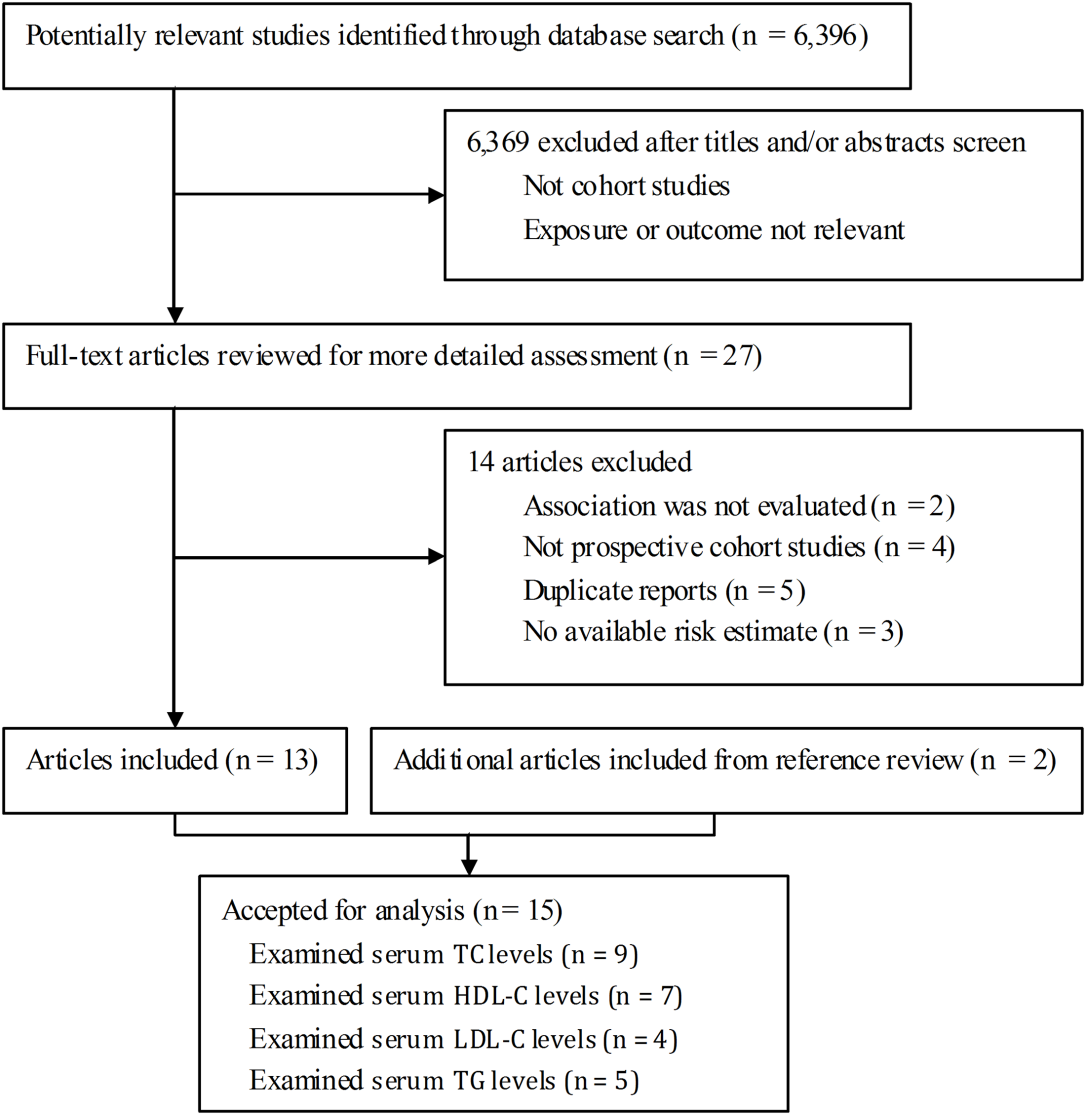
Flow diagram of study selection. Flow chart shows literature search and selection for prospective cohort studies of serum lipids in relation to breast cancer risk. TC = total cholesterol; HDL-C = high-density lipoprotein cholesterol; LDL-C = low-density lipoprotein cholesterol; TG = and triglycerides.

The detailed procedure of our literature search and selection is shown in Fig 1. Briefly, we identified 27 potentially relevant articles for full-text review. Of these, two articles were excluded because the association of interest was not evaluated [30, 31]. Four were not included because they used a retrospective cohort design or nested case-control design [32–35]. We also excluded five articles because they were duplicate reports [27, 36–39]. Three studies [40–42] were excluded due to lack of sufficient data to calculate the risk estimates. Two additional articles were included following a review of the reference lists. Altogether, 15 studies were included in this meta-analysis [5–19].

### Study characteristics

The main characteristics of the selected studies are summarized in Table 1. The 15 prospective cohort studies were published between 1992 and 2014. Four, eight, and three were conducted in the United States, Europe, and Asia, respectively. Among 1,189,635 participants, 23,369 cases of breast cancer were documented during follow-up periods ranging from 4 to 26 years. The majority of articles were population-based; two articles were conducted in nurses and teachers [8, 14]. Of the 15 included studies, 9 provided results for TC, 7 for HDL-C, 6 for TG, and 4 for LDL-C. One study [10] only included postmenopausal women only, and 6 of the other studies presented the estimates by menopausal status [7–9, 14, 16, 19]. The assessments of serum lipid profiles varied across studies, with most based on measurements using enzymatic methods. All studies identified cases through cancer registries or medical records. The adjusted covariates differed in individual studies, and most risk estimates were adjusted for age, body mass index, and smoking status. The results of study quality assessment (score 0–9) ranged from 6 to 9, with an average score of 7.8, indicating high quality.

**Table 1:** Characteristics of 15 prospective cohort studies of serum lipids and breast cancer included in this meta-analysis.

Author, year	Location, period	Ethnicity	Age(years), Menopausal status	Follow-up (years)	No. of cases/ participants	Exposure details	Comparison*	Adjusted RR (95% CI)	Study quality^†^	Adjustments
Hoyer et al, 1992	Danish, 1964–1989	Caucasian	30–80, overall	4–26	51/5207	TC	Q4 vs. Q1	1.0 (0.4–2.2)	8	Age, BMI, smoking, menopausal status, age at menarche, number of full-term, pregnancies, alcohol and coffee consumption
						LDL-C	Q4 vs. Q1	1.9 (0.5–6.6)		
						HDL-C	Q4 vs. Q1	0.3 (0.1–0.8)		
						TG	Q4 vs. Q1	1.9 (0.8–4.5)		
Gaard et al, 1994	Norwegian, 1977–1990	Caucasian	20–54, overall	10.4	302/31209	LDL-C	≥4.72 vs. <3.23 mmol/l	0.93 (0.67–1.29)	9	Age, BMI, height, menopausal status, smoking
Furberg et al, 2004	Norwegian, 1977–1998	Caucasian	17–54, overall	17.2	708/38823	HDL-C	>1.64 vs. <1.20 mmol/l	1.44 (0.91,2.30)^a^	9	Age, BMI, county of residence, parity, height, serum TC, physical activity, blood pressure, serum TG, age at first birth, time since last meal, smoking, energy and fat intake, menopausal status
								0.75 (0.58,0.97)^b^		
Eliassen et al, 2005	United States, 1990–2000	Caucasian	42–69, overall	<10	3177/71921	TC	≥6.21 vs. <4.65 mmol/l	0.94 (0.54–1.64)^a^	6	Age, BMI, age at menarche, parity, age at first birth, height, family history of breast cancer and BBD, alcohol consumption, physical activity, menopausal status, age at menopause, HRT use
								1.04 (0.91–1.17)^b^		
Kucharska et al, 2008	United States, 1987–2000	Caucasian	45–64, overall	NA	359/7575	HDL-C	>1.73 vs. <1.16 mmol/l	0.95(0.66–1.37)	9	Age, race, BMI, age at menarche, smoking, HRT use, age at menopause
Kabat et al, 2009	United States, 1993–2005	Caucasian	50–79, Postmenopause	8	165/4888	HDL-C	>1.62 vs. <1.29 mmol/l	0.80(0.53–1.20)	6	Age, education, race, BMI, oral contraceptive use, HRT use, age at menarche, age at first birth, age at menopause, alcohol, family history of breast cancer, history of breast biopsy, physical activity, energy intake, smoking, randomization of HRT, calcium plus vitamin D, and dietary modification trials, waist circumference, glucose, blood pressure
						TG	≥1.69 vs. <1.17 mmol/l	1.22 (0.82–1.80)		
Inoue et al, 2009	Japan, 1993–2004	Asian	40–69, overall	10.2	120/18176	HDL-C	≥1.03 vs. <1.03 mmol/l	1.54(0.98–2.44)	8	Age, study area, smoking, ethanol intake, serum TC
						TG	≥1.69 vs. <1.69 mmol/l	0.97(0.61–1.55)		
Iso et al, 2009	Japan, 1990–2004	Asian	40–69, overall	12.4	178/21685	TC	≥6.21 vs. <4.14 mmol/l	0.92(0.50–1.70)	9	Age, BMI, smoking, hypertension, diabetes, hyperlipidemia medication use, intake of total vegetable, coffee and ethanol, public health center
Bjorge et al, 2010	European, 1974–2005	Caucasian	≥29, overall	11	4862/287320	TG	Q5 vs. Q1	0.92 (0.76–1.11)	8	Age, BMI, year of birth, smoking, glucose
Fagherazzi et al, 2010	France, 1990–2005	Caucasian	40–65, overall	12	2932/69088	TC	>6.6 vs. ≤6.6 mmol/l	0.99 (0.85–1.15)	6	Age, intake of alcohol, total fat, and energy, Oral contraceptives use, age at menarche, age at menopause, number of children, age at first pregnancy, family history of breast cancer, history of BBD, diabetes status, education, HRT use
Kitahara et al, 2011	Korea, 1992–2006	Asian	30–95, overall	12.7	3805/433115	TC	≥6.21 vs. <4.4 mmol/l	1.17 (1.03–1.33)	8	Age, BMI, smoking, alcohol intake, glucose, hypertension, physical activity
Bosco et al, 2012	United States, 1995–2007	African	21–69, overall	10.5	1228/49172	TC	high vs. low	1.03 (0.90–1.17)	7	Age, BMI, education, physical activity, obesity, Type 2 diabetes, hypertension
							(self-reported)			
Melvin et al, 2012	Swedish, 1970–1996	Caucasian	≥25, overall	8.3	6105/234494	TC	≥6.30 vs. <4.80 mmol/l	0.97 (0.89–1.05)	7	Age, parity, level of glucose, TG, TC, fasting status, and socioeconomic status
						LDL-C	≥4.14 vs. <2.72 mmol/l	0.92 (0.75–1.13)		
						HDL-C	≥1.98 vs. <1.45 mmol/l	1.05 (0.86–1.29)		
						TG	≥1.30 vs. <0.70 mmol/l	0.91 (0.84–0.99)		
Strohmaier et al, 2013	European, 1972–2006	Caucasian	40.3–47.5(mean), overall	11.7	5228/288057	TC	Q5 vs. Q1	0.70 (0.61–0.81)	8	Age, BMI, smoking
His et al, 2014	France, 1994–2007	Caucasian	case: 49.5±6.1(mean), overall	11.5	141/4433	TC	≥6.54 vs. <5.02 mmol/l	0.65 (0.39–1.10)	9	Age, BMI, intervention group, number of dietary records, alcohol intake, physical activity, smoking, education, height, family history of breast cancer, menopausal status, number of full-term, HRT use, energy intake, hyperlipidemia medication use, glycaemia
						LDL-C	≥4.11 vs. <3.11 mmol/l	0.65 (0.39–1.09)		
						HDL-C	≥2.07 vs. <1.66 mmol/l	0.60 (0.36–1.01)		
						TG	≥1.06 vs. <0.58 mmol/l	0.97 (0.57–1.65)		

Abbreviations: BMI, body mass index; HRT, hormone replacement therapy; BBD, benign breast disease; Q, quintile; RR, relative risk; CI, confidence interval; NA, not available; TC, total cholesterol; LDL-C, low-density lipoprotein cholesterol; HDL-C, high-density lipoprotein cholesterol; TG, Triglycerides

* The highest vs lowest level of exposures

^a^ Premenopause,

^b^ Postmenopause

^†^ According to the Newcastle-Ottawa Quality Assessment Scale for cohort studies

### Main analysis

#### TC

**Fig 2:**
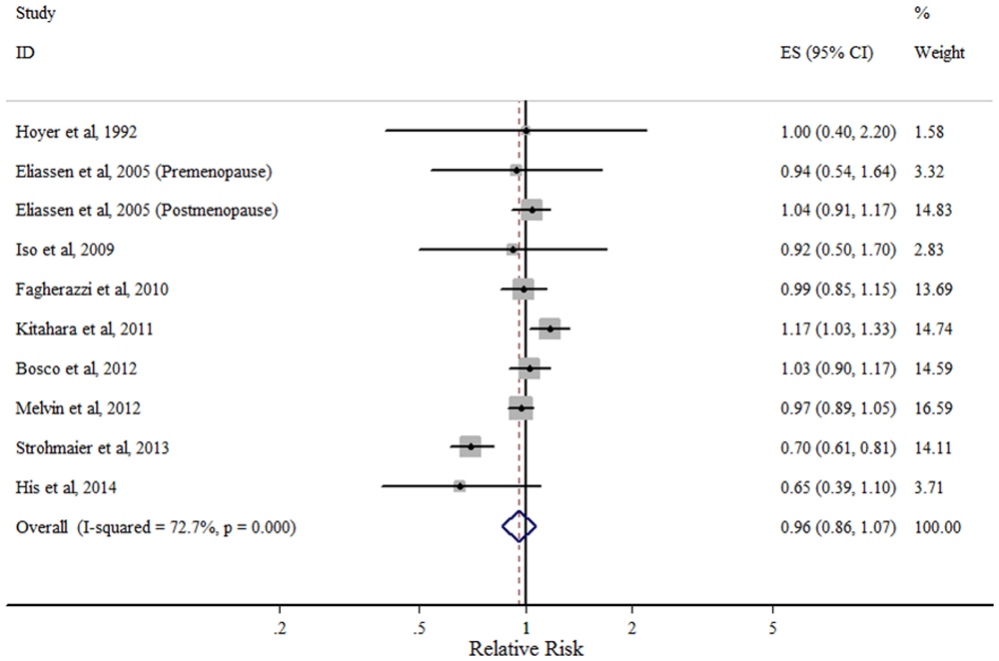
Forest plot of the highest vs. lowest categories of serum TC levels and breast cancer risk. Squares indicate study-specific relative risk estimates (size of the square reflects the study-specific statistical weight); horizontal lines indicate 95% confidence intervals (CI); diamond indicates the overall relative risk with its 95% confidence interval.

The multivariable-adjusted RRs for each study and the combined RR for the highest versus lowest TC categories are shown in Fig 2. Results from nine included studies were inconsistent, with only two of them reaching statistical significance. The summary RR comparing the highest and lowest level of TC was 0.96 (95% CI: 0.86–1.07). There was considerable heterogeneity across studies (*P*_heterogeneity_ = 0, *I* ^2^ = 72.7%).

#### HDL-C

**Fig 3:**
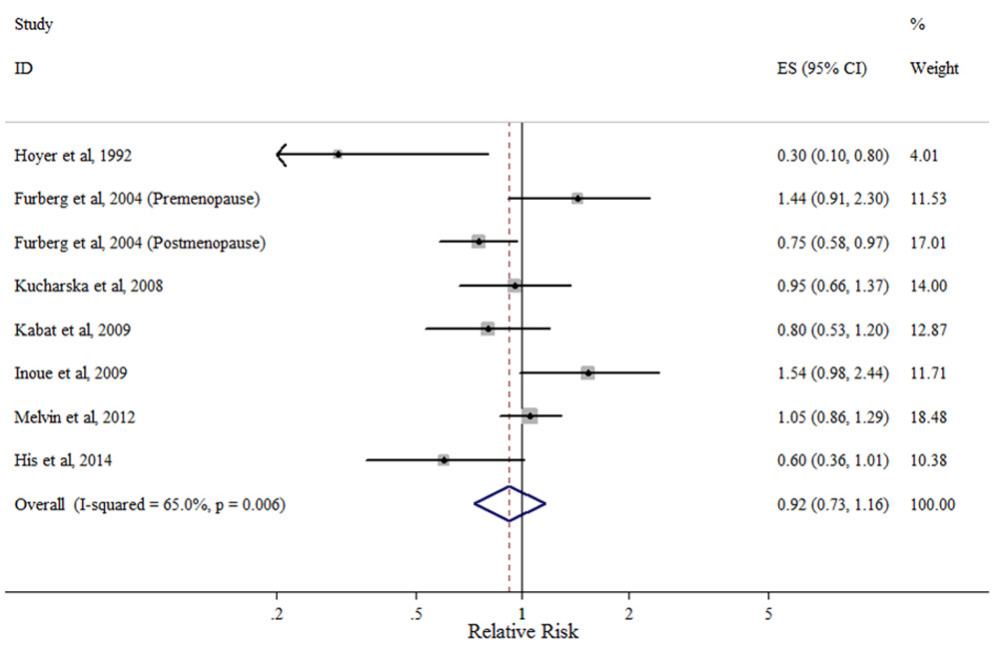
Forest plot of the highest vs. lowest categories of serum HDL-C levels and breast cancer risk. Squares indicate study-specific relative risk estimates (size of the square reflects the study-specific statistical weight); horizontal lines indicate 95% confidence intervals (CI); diamond indicates the overall relative risk with its 95% confidence interval.

Seven studies presented results on the highest versus lowest levels of HDL-C and breast cancer risk (Fig 3). The majority of included studies reported a negative association, and two found a significant correlation [5, 7]. The summary risk estimate of breast cancer for the highest TG compared with the lowest was 0.92 (95% CI: 0.73–1.16), with evidence of substantial heterogeneity (*P*_heterogeneity_ = 0.006, *I* ^2^ = 65.0%).

#### LDL-C

**Fig 4:**
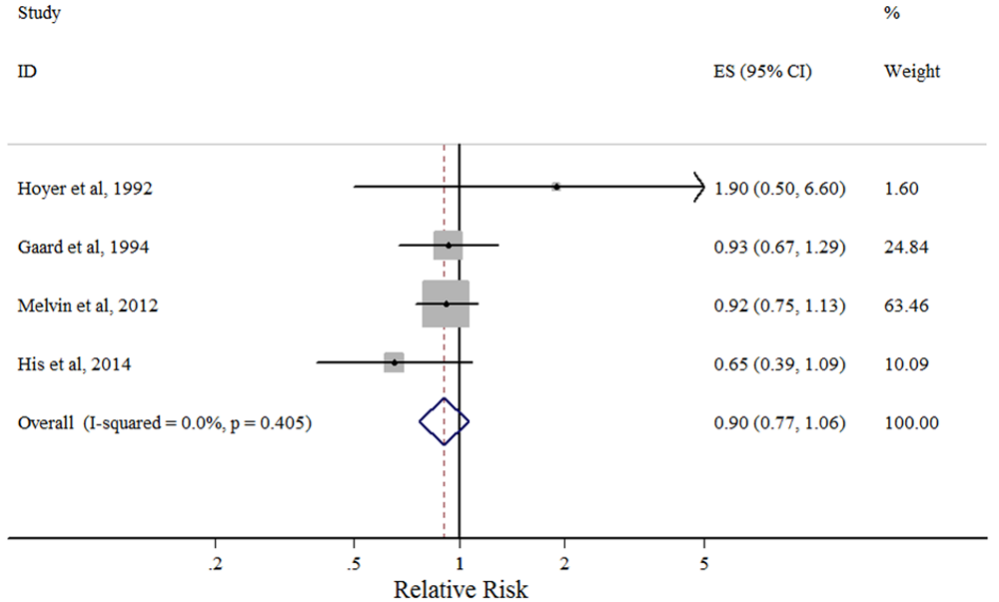
Forest plot of the highest vs. lowest categories of serum LDL-C levels and breast cancer risk. Squares indicate study-specific relative risk estimates (size of the square reflects the study-specific statistical weight); horizontal lines indicate 95% confidence intervals (CI); diamond indicates the overall relative risk with its 95% confidence interval.

Four studies analyzed the role of LDL-C in breast cancer risk. The results are shown in Fig 4. None of the individual studies reported significant associations. The combined RR for the highest versus lowest LDL-C concentrations was 0.90 (95% CI: 0.77–1.06). There was no indication of heterogeneity (*P*_heterogeneity_ = 0.405, *I* ^2^ = 0.0%).

#### TG

**Fig 5:**
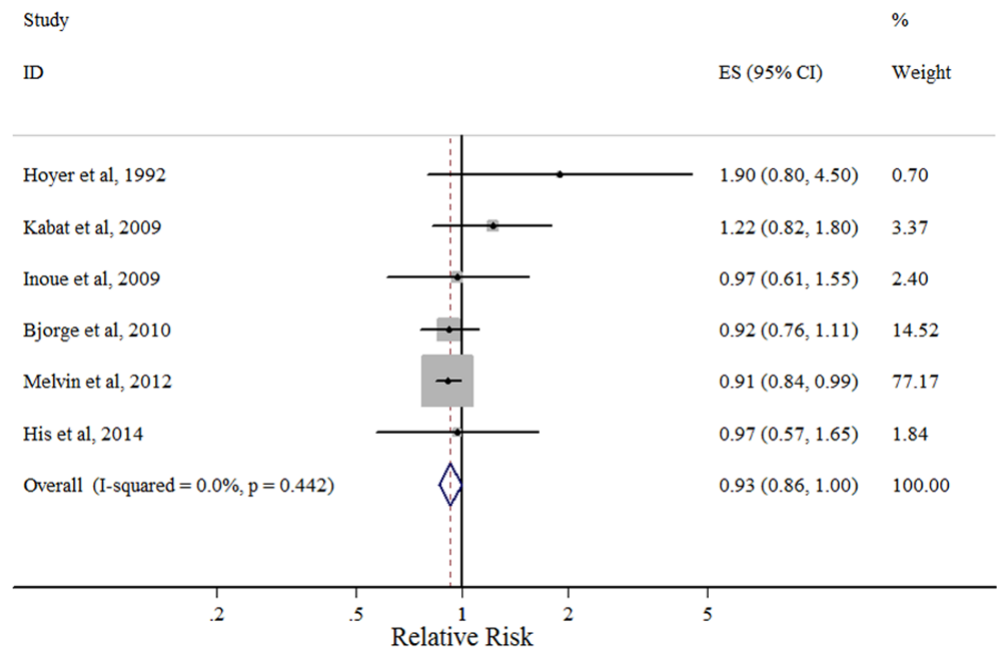
Forest plot of the highest vs. lowest categories of serum TG levels and breast cancer risk. Squares indicate study-specific relative risk estimates (size of the square reflects the study-specific statistical weight); horizontal lines indicate 95% confidence intervals (CI); diamond indicates the overall relative risk with its 95% confidence interval.

An association between breast cancer risk and serum TG was reported in six studies. Fig 5 shows the forest plots for the highest versus lowest TG categories. Of the six eligible studies, four reported an inverse relation, whereas only one reached statistical significance. Compared with the lowest category, the pooled RR for the highest category was 0.93 (95% CI: 0.86–1.00), with no evidence of heterogeneity among studies (*P*_heterogeneity_ = 0.442, *I* ^2^ = 0.0%).

### Subgroup and sensitivity analyses

The results of subgroup analyses according to geographic region, ethnicity, follow-up length, number of cases, menopausal status, and adjustment for dietary factors are presented in Table 2. We found no evidence of effect modification for TC in most subgroups, except when stratifying by geographic region and ethnicity. Notably, a significantly positive relation between TC and breast cancer risk was detected among studies in Asian populations (RR = 1.16, 95% CI: 1.02–1.31). The majority of strata did not materially change the association between HDL-C and breast cancer risk; however, when we stratified the analysis according to menopausal status, an inverse association between HDL-C and breast cancer risk was observed among women who were postmenopausal at baseline (RR = 0.77, 95% CI: 0.64–0.93) but was not statistically significant in premenopausal women (RR = 0.84, 95% CI: 0.40–1.74). Concerning TG, a significantly decreased risk of breast cancer was observed among studies in Caucasians and those with ≥300 subjects that unadjusted for dietary factors.

**Table 2:** Stratified meta-analyses of three lipid components and breast cancer risk.

Group	TC				HDL-C				TG			
	No. of studies	RR (95% CI)	*P*_heterogeneity_	*I* ^2^, %	No. of studies	RR (95% CI)	*P*_heterogeneity_	*I* ^2^, %	No. of studies	RR (95% CI)	*P*_heterogeneity_	*I* ^2^, %
Total	9	0.96(0.86–1.07)	0	72.7	7	0.92(0.73–1.16)	0.006	65.0	6	0.93(0.86–1.00)	0.442	0
Geographic area												
United States	2	1.03(0.94–1.13)	0.940	0	2	0.88(0.67–1.16)	0.539	0	1	1.22(0.82–1.80)	-	-
Europe	5	0.86(0.71–1.03)	0.001	78.1	4	0.84(0.60–1.17)	0.005	72.8	4	0.92(0.85–0.99)	0.422	0
Asia	2	1.16(1.02–1.31)	0.451	0	1	1.54(0.98–2.43)	-	-	1	0.97(0.61–1.55)	-	-
Ethnicity												
Caucasian	6	0.90(0.78–1.04)	0.001	72.7	6	0.86(0.69–1.08)	0.019	60.4	5	0.93(0.86–1.00)	0.313	15.9
Asian	2	1.16(1.02–1.31)	0.451	0	1	1.54(0.98–2.43)	-	-	1	0.97(0.61–1.55)	-	-
African	1	1.03(0.90–1.17)	-	-	-	-	-	-	-	-	-	-
Length of follow-up												
< 10 y	2	0.99(0.92–1.06)	0.651	0	2	0.97 (0.77–1.24)	0.243	26.8	2	0.99(0.76–1.28)	0.153	51.1
≥ 10 y	6	0.92(0.76–1.12)	0	84.2	3	0.99 (0.64–1.53)	0.004	77.9	3	0.93(0.79–1.10)	0.967	0
No. of cases												
< 300	3	0.79(0.55–1.13)	0.584	0	4	0.77(0.44–1.33)	0.007	75.4	4	1.13(0.88–1.45)	0.519	0
≥ 300	6	0.97(0.86–1.10)	0	80.3	3	0.98(0.78–1.24)	0.065	58.5	2	0.91(0.85–0.98)	0.917	0
Menopausal status												
Premenopause	3	0.99(0.84–1.17)	0.832	0	3	0.84(0.40–1.74)	0.036	70	-	-	-	-
Postmenopause	3	1.05(0.96–1.14)	0.657	0	4	0.77(0.64–0.93)	0.391	0.1	-	-	-	-
Adjustment for dietary factors												
Yes	6	1.05(0.96–1.14)	0.323	14.0	5	0.87(0.61–1.23)	0.003	72.3	4	1.13(0.88–1.45)	0.519	0
No	3	0.89(0.72–1.10)	0	89.3	2	1.03(0.86–1.22)	0.639	0	2	0.91(0.85–0.98)	0.917	0

To assess any impact of study quality on the effect estimates, we performed a sensitivity analysis that only included high-quality studies. The combined risk estimates did not show any substantial difference compared to the overall results. Furthermore, we conducted sensitivity analyses in which one study was removed and the rest were analyzed. The results showed that substantial heterogeneity in TC and breast cancer association was most likely due to the Strohmaier et al study [18]; excluding it resulted in a homogenous but still non-significant result (RR = 1.02, 95% CI: 0.96–1.08, *P*_heterogeneity_ = 0.33, *I* ^2^ = 13.0%). The influence of each individual data set on the combined risk estimates was not significant for the other lipid components.

### Dose-response analysis

Overall, we failed to identify a significant dose-response relationship between any lipid components and breast cancer risk using data from five studies on TC [6, 12, 15, 19, 27], three studies on HDL-C [6, 9, 19], and three studies on TG [6, 19, 27]. For each 1 mmol/l increase, the summary RRs of breast cancer were 0.99 (95% CI: 0.95–1.04, *P*_heterogeneity_ = 0.20) for TC, 0.96 (95% CI: 0.88–1.04, *P*_heterogeneity_ = 0.38) for HDL-C, and 0.95 (95% CI: 0.84–1.07, *P*_heterogeneity_ = 0.56) for TG.

### Publication bias

There was no evidence of publication bias for individual lipid components based on Begg’s rank correlation and Egger linear regression tests (all *P* > 0.05).

## Discussion

The present meta-analysis of 15 prospective cohort studies involving 23,369 cases among 1,189,635 participants showed that serum lipid levels were inversely associated with the risk of breast cancer, but only TG reached statistical significance. Subgroup analyses revealed that HDL-C was related to a significantly reduced risk of breast cancer among postmenopausal but no premenopausal women. In further analyses of dose-response models, similar trends between lipid components and overall breast cancer risk were detected but were not significant.

We observed substantial heterogeneity among studies regarding the associations of TC and HDL-C with breast cancer risk. This is not surprising given the differences in study designs, population characteristics, follow-up lengths, as well as selection of analysis covariates. Our sensitivity analyses suggested that the observed heterogeneity in TC and breast cancer association seemed to be explained by one large cohort study among participants from Norway, Austria, and Sweden [18]. After exclusion of this single study, there was a still non-significant association between TC levels and risk of breast cancer without evidence of study heterogeneity. The disparate result for this European cohort study may be due to their lack of controlling for potential confounding factors (e.g., physical activity, alcohol consumption, and endogenous hormone levels [43]) compared with the other studies (Table 1).

In our analysis by ethnicity, we observed a significantly positive association between TC and breast cancer risk in Asian but not in Caucasian populations, indicating that ethnicity difference might also be a potential source of heterogeneity. However, we cannot deny the possibility that the racial disparity was a chance finding because only two studies [12, 15] were involved in the Asian subanalysis. More studies are needed to determine whether this association is valid.

With regard to HDL-C, our subgroup analysis suggested that menopausal status likely contributed to the substantial across-study heterogeneity. It is interesting that there was a significantly reduced risk of postmenopausal but not premenopausal breast cancer with elevated HDL-C levels, suggesting that menopause may modify the relationship between HDL-C and breast cancer risk. In line with our finding, one recent meta-analysis that assessed the impact of metabolic syndrome on postmenopausal breast cancer risk also observed a protective effect of serum HDL-C, but they did not assess the same affect for premenopausal breast cancer risk [20]. Moreover, two of the three included studies had a retrospective design, rendering them prone to systematic bias. Interpreting our observation of the differences by menopausal status is challenging. Perhaps it could be explained in the context of hormonal regulation of breast cancer. Indeed, serum HDL-C levels have been regarded as a marker of androgen status [44]. After menopause, the aromatization of androgens to biologically active estrogens within adipose tissue plays a major role in breast carcinogenesis [45]. In this regard, low HDL-C might reflect a relative androgen deficit and serve as a clue for the possible development of breast cancer among postmenopausal women. Therefore, our result of a null association between serum HDL-C and overall breast cancer incidence should not be overemphasized, and further research evaluating this association should take menopausal status into account.

It was reported that dietary is an important manager of both serum lipid levels [46] and breast cancer risk [47]. In this meta-analysis, most of eligible studies (9 of 15) adjusted for major dietary factors, including total dietary fat, energy intake, vegetable consumption, and coffee and alcohol intake. In addition, our stratified analyses showed that dietary factors did not appear to have a substantial impact on the associations with serum lipids except TG. There was no significant relationship between serum TG and breast cancer risk when the analysis was restricted to studies that adjusted for dietary factors. However, this result should be interpreted with caution because the number of cases was extremely limited (from 11444 to 447) in the current analysis.

Several explanations can be advanced for the inverse associations observed between dyslipidemia and breast cancer risk. The beneficial effect of HDL-C was proposed to be related to its anti-oxidative and anti-inflammatory properties. Experimental studies have suggested that HDL-C can prevent lipid peroxidation by inhibiting LDL-C oxidative damage [48, 49]. Moreover, increased serum HDL-C is associated with greater production of anti-inflammatory cytokines such as interleukin 10, which thought to play a protective role against breast cancer [50, 51]. HDL-C levels are also inversely associated with levels of insulin-like growth factor-I (IGF-I), which could increase the mammographic density of breast during postmenopausal years and promote carcinogenesis [52]. In contrast to HDL-C, the biological mechanisms underlying the inverse association between TG and breast cancer are less investigated and remains unclear. A plausible hypothesis is competing cardiovascular risk, by which deaths due to cardiovascular events is apt to increase with hypertriglyceridemia [53], thus reducing the proportion of breast cancer cases in subjects with elevated TG levels.

Lipid-lowering drugs such as statins are among the most commonly prescribed medications worldwide, but there is still a debate concerning their correlation with the risk of cancers including breast. Undela et al recently combined the results of 24 observational studies including more than 2.4 million participants and 76,759 cases and failed to detect any significant reduction of breast cancer incidence with either short or long-term statin use [54]. Indeed, the reduction of serum LDL-C is purported to be the main mechanism through which statins exert their effects. Therefore, our finding of a null association between serum LDL-C and breast cancer risk reinforced and partially explained the lack of efficacy of statins.

The major strength of this meta-analysis is that all included studies used a prospective cohort design, which eliminates the possibility of reverse causation (i.e., breast cancer itself may have altered serum lipid levels [55]) and minimizes systematic bias. Besides, the large number of total participants and high-quality studies provided sufficient statistical power to detect relatively modest effects and draw credible conclusions.

However, several limitations of our study should also be acknowledged. First, given the non-randomization design, residual confounders were of concern. We were unable to rule out potential confounding due to unmeasured variables such as family history of other types of cancer, which could exaggerate or obscure the true associations. Moreover, the adjusted covariates, for instance dietary factors (e.g., intake of fat, energy, vegetable, coffee, and alcohol), differed in individual studies, which might also increase the risk of confounding bias. Second, we observed substantial heterogeneity that could have been introduced by clinical or methodological differences among studies, including variation in geographic regions, menopausal status, number of cases, follow-up length, and analysis covariate selection. We determined that across-study heterogeneity for serum TC and HDL-C seemed was largely driven by one European cohort study [18] that did not make important adjustments and subjects with different menopausal status, respectively. There was also a wide difference in cutoff points for the lowest and highest categories, which might have also contributed to between-study variation. However, we do not believe this dramatically impacted our findings because the present dose-response assessments, which eliminated the bias of divergent cutoff criteria, obtained similar results as the highest versus lowest concentration analyses. Third, ethnic differences could play an important role in breast cancer pathogenesis. Most of the eligible studies included in this meta-analysis were carried out in Caucasian populations, and we cannot exclude the possibility that different associations, especially for TC, exist among Asians. Fourth, concerning TG, we were unable to perform separate analysis of pre- and postmenopausal breast cancer due to the lack of data stratification by menopausal status. To date, only one prospective cohort study has investigated the relationship between TG and postmenopausal breast cancer risk and found no association [10]. Notably, a previous meta-analysis found no effect of TG on postmenopausal breast cancer risk [20], whereas we saw a protective role in total breast cancer occurrence, suggesting that menopausal status might be a potential modifier. Fifth, because lipid levels are not isolated other markers of dyslipidemia, including the ratio of TC to HDL-C, LDL-C to HDL-C, and TG to HDL-C, merit consideration. However, due to the lack of available data, we could not investigate associations between cholesterol ratios and breast cancer risk. Finally, publication bias is a concern in any meta-analysis. Although Begg’s and Egger’s tests revealed no evidence of such a bias, the analyses were underpowered because of the relatively small number of included studies.

In summary, the results of this meta-analysis indicate that elevated serum levels of TG may be associated with a reduced breast cancer risk. Among postmenopausal women, serum HDL-C may also play a protective role against breast carcinogenesis. We did not find significant associations between breast cancer risk and serum TC and LDL-C concentrations. Future research with larger sample sizes, detailed menopausal status information and consistent adjustments for confounders, especially among Asian populations, is warranted to extend our findings.

## Supporting Information

S1 File Literature search strategy. (DOC)

S1 PRISMA Checklist PRISMA 2009 Checklist. (DOC)
