# Supplementary material for: Serum Lipids and Breast Cancer Risk: A Meta-Analysis of Prospective Cohort Studies
Source: PLoS One. 2015 Nov 10;10(11):e0142669. doi: 10.1371/journal.pone.0142669 (PMC4640529; doi:10.1371/journal.pone.0142669)
Supplement: S1 File — (DOC) [file pone.0142669.s001.doc]

**Literature Search Strategy**

**PubMed**

(("breast neoplasms"[MeSH Terms] OR ("breast"[All Fields] AND "neoplasms"[All Fields]) OR "breast neoplasms"[All Fields] OR ("breast"[All Fields] AND "cancer"[All Fields]) OR "breast cancer"[All Fields]) AND ((((("lipids"[MeSH Terms] OR "lipids"[All Fields] OR "lipid"[All Fields]) OR ("lipoproteins"[MeSH Terms] OR "lipoproteins"[All Fields] OR "lipoprotein"[All Fields])) OR ("cholesterol"[MeSH Terms] OR "cholesterol"[All Fields])) OR ("triglycerides"[MeSH Terms] OR "triglycerides"[All Fields] OR "triglyceride"[All Fields])) OR ("dyslipidemias"[MeSH Terms] OR "dyslipidemias"[All Fields] OR "dyslipidemia"[All Fields]))) AND ((("risk"[MeSH Terms] OR "risk"[All Fields]) OR ("epidemiology"[Subheading] OR "epidemiology"[All Fields] OR "incidence"[All Fields] OR "incidence"[MeSH Terms])) OR ("epidemiology"[Subheading] OR "epidemiology"[All Fields] OR "prevalence"[All Fields] OR "prevalence"[MeSH Terms]))

**Embase**

((('breast neoplasms'/exp OR 'breast neoplasms') OR ('breast cancer'/exp OR 'breast cancer')) AND (('lipid'/exp OR lipid) OR ('lipoprotein'/exp OR lipoprotein) OR ('cholesterol'/exp OR cholesterol) OR ('triglyceride'/exp OR triglyceride) OR ('dyslipidemia'/exp OR dyslipidemia)) AND (('risk'/exp OR risk) OR ('incidence'/exp OR incidence) OR ('prevalence'/exp OR prevalence)))
